# Effects of Bed-Rest on Urea and Creatinine: Correlation with Changes in Fat-Free Mass

**DOI:** 10.1371/journal.pone.0108805

**Published:** 2014-09-29

**Authors:** Giancarlo Bilancio, Cinzia Lombardi, Rado Pisot, Natale G. De Santo, Pierpaolo Cavallo, Massimo Cirillo

**Affiliations:** 1 Department of Medicine and Surgery, University of Salerno, Baronissi, Italy; 2 Department of Maternity and Pediatrics, Hospital of Benevento, Benevento, Italy; 3 Institute for Kinesiology Research, University of Primorska, Koper, Slovenia; 4 Department of Medicine, Second University of Naples, Naples, Italy; 5 Department of Physics, University of Salerno, Fisciano, Italy; West Virginia University School of Medicine, United States of America

## Abstract

**Background:**

Bed-rest experiments are designed for investigation on catabolic effects of hypokinetic conditions and/or for microgravity simulation in on-ground aerospace research. Bed-rest effects include a reduction in fat-free mass and muscle mass. Urea and creatinine are catabolites of endogenous protein and of muscular energetic metabolism which are excreted mainly by the kidney. The study investigated on urea, creatinine, and kidney function during bed-rest.

**Methods:**

Twenty healthy young men underwent a 7-day adaptation period (day-6 to day-0) and a 35-day bed-rest experiment (day1 to day35) during normocaloric diet. Urine were collected from day-3 to day0 (baseline) and from day1 to day35. Blood samples and anthropometrical data were collected at day0 (baseline) and bed-rest days 7, 14, 21, 28, and 35.

**Results:**

Bed-rest reduced plasma volume, weight, fat-free mass, and muscle mass (P<0.001). During bed-rest there was a transient increase in plasma and urinary urea, a decrease in plasma creatinine, and no change in urinary creatinine. The overall integral of changes from day0 to day35 was on average +101.7 mg/dL for plasma urea (95%CI = +43.4/+159.9), +82.2 g/24 h for urinary urea (95%CI = +55.8/+108.7), −2.5 mg/dL for plasma creatinine (95%CI = −3.1/−1.9). Bed-rest reduced plasma cistatyn C also, which was used as mass-independent marker of glomerular filtration rate (−13.1%, P<0.05). Correlations with final reduction in fat-free mass and muscle mass were significant for the overall integral of changes in urinary urea from day0 to day35 (R = 0.706, P<0.001) and for early changes in urinary urea and plasma urea from day0 to day7 (R = 0.566, P = 0.009 and R = 0.715, P<0.001, respectively).

**Conclusions:**

Study results shows that urea is a marker of catabolic conditions secondary to hypokinetic conditions.

## Introduction

Experiments based on a rigorous regimen of continuous bed-rest (bedrest) are performed for investigation about the effects of hypokinetic conditions during hospitalization and/or for simulation of microgravity on Earth in aerospace medical research [1], [2]. The effects of bedrest are various and include the reduction of plasma volume, bone mass, and skeletal muscle mass [2]–[5]. A reduction in muscle mass could theoretically be associated with changes in the metabolism of urea and creatinine. With regard to urea, a muscle mass reduction could increase the urea generation because the muscular tissue has a high protein content and urea is the final catabolite of endogenous protein breakdown [6]. With regard to creatinine, skeletal muscle mass is the main determinant of creatinine generation because creatinine is the final catabolite of muscular energetic metabolism [7]. Available data are limited or inconsistent about urea and creatinine metabolism during bedrest [8]–[13]. Previous bedrest studies reported for urinary urea either an increase [8]–[11] or no change [11]–[13] and no effect for urinary creatinine [9]. None of these previous studies addressed the possible confounding of changes in kidney function during bedrest although it is known that the kidneys account for about 70% of urea excretion [6] and for almost all of creatinine excretion [7]. Moreover, research data are missing also about the possible correlation of changes in urea or creatinine levels with changes in muscle mass during bedrest.

Therefore, the present study was designed for investigations on urea and creatinine levels during bedrest with control for possible changes in kidney function and with main focus on two objectives: the time course of changes in plasma/urine levels of urea and creatinine and the use of these changes as marker or predictor of changes in body mass.

## Methods

Two 35-day bedrest experiments were performed in the Valdoltra Orthopedic Hospital (Slovenia) as part of the OSMA project (OSteoporosis and Muscular Atrophy), a collaborative study which was sponsored by the Italian Space Agency (Agenzia Spaziale Italiana, ASI) [14]–[18]. The funder had no role in study design, data collection and analysis, decision to publish, or preparation of the manuscript. The study protocols were identical in the two experiments except for the use of horizontal bedrest in the first experiment (0 degrees) and of anti-orthostatic head-down bedrest in the second experiment (−6 degrees). The experiments conformed to the Declaration of Helsinki and were approved by the Slovenian National Committee for Medical Ethics at the Ministry of Health (Republic of Slovenia). Twenty healthy men participated in the two experiments after the release of the informed written consent (10 men for each experiment). Selection criteria were stable body mass in the last three months, absence of musculoskeletal disorders, absence of significant disorders at routine laboratory investigation and medical examination. The bedrest period was preceded by 7-day ambulatory adaptation within the premises of the Hospital (day-6 to day0) followed by 35 days of continuous bedrest (day1 to day35). Physical activity was not permitted at any time during bedrest. Muscular contractures were prevented by passive joint mobilization, which was performed three times weekly by a qualified physiotherapist as reported [15]. Normocaloric diets were individually tailored on the basis of the resting energy expenditure derived from the reference standards of the World Health Organization [19]. Dietary energy requirements were designed as 1.4 the resting expenditure during ambulatory adaptation and as 1.2 the resting expenditure during bedrest, respectively [14]–[18]. The two dietary regimens were qualitatively identical including six daily meals (breakfast, lunch, dinner, and three snacks) and containing the following percentages (%) of total calories: carbohydrates = 60%, fats = 25%, and proteins 15%. Twenty-four-hour urine was accurately collected in the last four days of the adaptation week (from day-3 to day0) and in each one of the 35 days of bedrest (from day1 to day35). Venous blood samples were collected early in the morning after an overnight fast in the last day of the adaptation week and weekly during bedrest (day0 and days 7, 14, 21, 28, and 35). At the same days, data collection included the morning measurements of weight and bioelectric impedance analysis for the calculation of fat mass, fat-free mass, and skeletal muscle mass (Akern, Florence, Italy). Bone scans were obtained by peripheral quantitative computer tomography at day0 and day35 (Stratec Medizintechnik, Pforzheim, Germany) [15]. Adherence to the protocol was ascertained by nurses surveillance, 24 h video surveillance, and medical supervision. Adherence to dietary prescriptions was ascertained by daily food records collected by trained dieticians.

Lab measurements included urinary urea, plasma urea, urinary creatinine, plasma creatinine, plasma cystatin C, and blood count. Urea, creatinine, and cystatin C were measured by automated biochemistry with the use of commercially available kits (Roche, Basel, Switzerland). Technical error in blind duplicates was <10% of mean for all lab measurements.

Bioimpedance data were used to asses if changes in weight reflected changes in fat mass, fat-free mass, and skeletal muscle mass [20]. Fat mass maintenance was used as index of energy balance. Changes in kidney function were assessed by plasma creatinine and plasma cystatin C [21]. Plasma cystatin C was included as a muscle mass-independent index of glomerular filtration [21]. Changes in hemoglobin and hematocrit were used to estimate changes in plasma volume [22], [23].

### Calculations and statistics

Changes during bedrest were calculated as the value at given day of bedrest minus the pre-bedrest value(s) defined as baseline. Baseline was the mean of the four pre-bedrest values for 24 h urinary variables (from day-3 to day0 included) and the single pre-bedrest value at day 0 for anthropometrical indices and for blood variables. As per this definition, positive changes indicated an increase over baseline whereas negative changes indicated a decrease below baseline. The integral of changes from day1 to day35 was used as overall measure of bedrest-induced changes in plasma and urinary variables. Bedrest effects were considered statistically significant if the 95% confidence interval (95%CI) of the integral of changes did not include zero. Simple and partial correlation analysis was used to assess if changes in plasma or urinary variables correlated with or predicted the after-bedrest final changes in anthropometrical indices. Statistical procedures included also t-test for paired data for comparisons between different time-points and ANOVA for comparison between data of two bedrest experiments.

## Results

**Figure 1:**
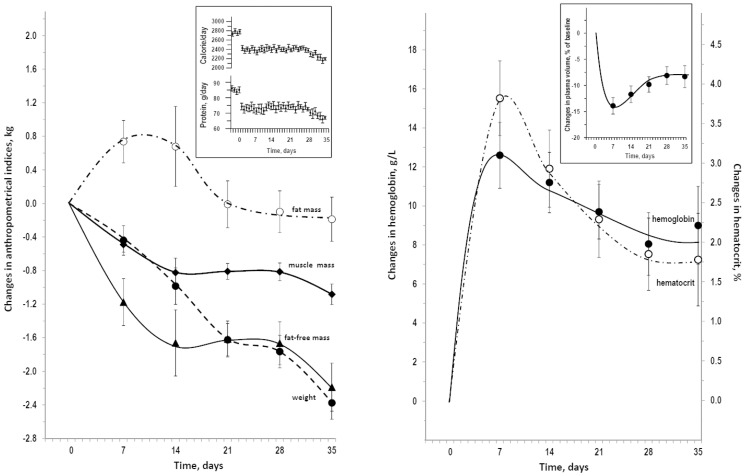
Dietary data, anthropometrics, and erythrocyte-related indices during bedrest. On the left, changes over baseline in anthropometrical indices and dietary data in the insert. On the right: changes over baseline in erythrocyte-related indices and estimated changes in plasma volume in the insert. Data are shown as mean±SEM. Differences versus day0 were statistically significant at day7 only for fat mass (P = 0.009), at all time-points from day7 to day35 for weight, fat-free mass, muscle mass, hemoglobin, and hematocrit (P≤0.022).

Table 1 reports the descriptive statistics at day0 and at day35. Mean±SEM was 23.8±0.5 for age and 179±2 cm for height. Data were similar in the subgroup of 10 participants in the horizontal bedrest and the subgroup of 10 participants in the head-down bedrest (not shown, P>0.374 in ANOVA for comparisons between subgroups). Figure 1 summarizes dietary data, changes in anthropometrical indices and in erythrocyte-related indices during bedrest. The after-bedrest decrease in weight was accounted for a decrease in fat-free mass because fat mass during bedrest overlapped baseline with exception of a transient increase at day7. The after-bedrest final change in fat-free mass averaged almost the same value of change in weight but correlated better with change in muscle mass (R = 0.796, P = 0.001) than with change in weight (R = 0.473, P = 0.035). Fat-free mass reduction during bedrest was not linear over time for being faster in the first 2 weeks as compared to the last two weeks (change from day0 to day14 vs change from day21 to day35: −1.66±0.39 vs −0.57 kg, P = 0.009). A similar difference was found for muscle mass reduction (−0.82±0.17 vs −0.27±0.11 kg, P = 0.010). The muscle mass/fat-free mass ratio was 0.524±0.015 at day0 and was stable throughout the bedrest. Findings were similar in separate analyses for horizontal bedrest and head-down bedrest (not shown). Hemoglobin and hematocrit had a parallel peak increase at day7 of bedrest followed by a stable above-baseline plateau after day21 (Figure 1). The estimates of plasma volume changes were an initial large decrease followed by a stable decrease about 8% below baseline (right insert in Figure 1). Hemoglobin changes were higher in head-down bedrest than horizontal bedrest at day7 (+16.4 and +8.8 g/L, F = 6.55, P = 0.020) but were not significantly different at day14 (+12.5 and +9.9 g/L, F = 0.69, P = 0.418) and were similar from day21 on (+10.5 and +9.9 g/L, F = 0.69, P = 0.418; +7.7 and +8.4 g/L, F = 0.05, P = 0.835). Findings were similar for hematocrit changes (not shown). Accordingly, the estimates of plasma volume changes were a larger decrease in head-down bed-rest at day7 but similar decreases not from day14 to day35 (not shown).

**Table 1:** Descriptive statistics at baseline and at bedrest termination (day0 and day35).

	Baseline (day0)		Bedrest termination (day35)	
	mean±SEM	min-max	mean±SEM	min-max
Weight, kg	73.7±2.2	54.2–98.1	71.4±2.1	52.9–94.6
Fat mass, kg	12.8±1.1	8.0–27.9	12.6±1.2	8.0–29.5
Fat-free mass, kg	60.9±1.4	45.4–75.2	58.7±1.3	44.5–73.2
Skeletal muscle mass, kg	31.9±0.8	24.4–39.8	30.8±0.7	23.9–39.6
Dietary calorie, calorie/day	2781±46	2347–3084	2194±31	1701–2588
Dietary protein, g/day	85.0±1.9	70–97	67.1±0.9	52–91
Hemoglobin, g/L	145.6±2.1	139–165	154.6±2.5	144–175
Hematocrit, %	43.2±0.7	41.5–49.4	45.0±0.8	41.9–50.6

### Urea and creatinine during bedrest

**Figure 2:**
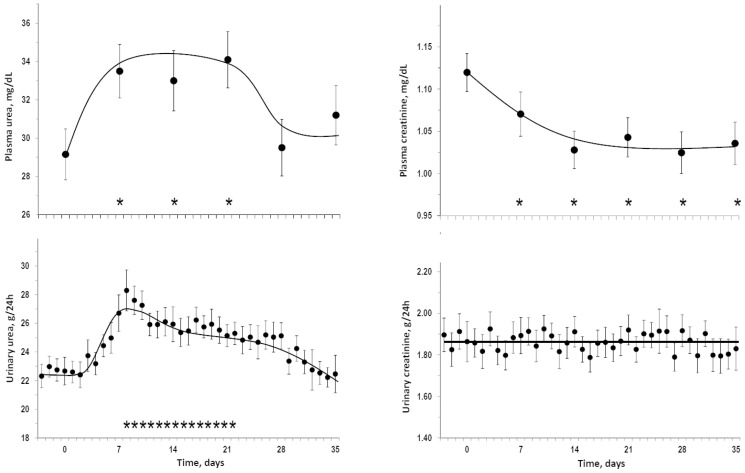
Urea and creatinine during bedrest. Mean±SEM pre-bedrest and during bedrest of plasma and urinary levels of urea and creatinine. Stars above the x-axis lines indicate time-points which were statistically different versus day0 (P<0.05).

Figure 2 summarizes data about plasma and urinary levels of urea and creatinine during bedrest. Approximately at day7 of bedrest there was a peak increase in the means of plasma and urinary urea followed by a trend toward a return to baseline values after day21 for plasma urea and after day28 for urinary urea. Plasma creatinine stably decreased from day7 on in the presence of negligible changes in urinary creatinine. Analyses of individual integrals of overall changes from day1 to day35 indicated that bedrest induced a significant increase in plasma urea (mean = +101.7 mg/dL, 95%CI = +43.4/+159.9), a significant increase in urinary urea (mean = +82.2 g, 95%CI = +55.8/+108.7), a significant decrease in plasma creatinine (mean = −2.5 mg/dL, 95%CI = −3.1/−1.9), but no significant change in urinary creatinine (mean = 2.9 g, 95%CI = −1.1/+3.6). Findings were similar in separate analyses for horizontal bedrest and head-down bedrest (not shown). As observed for plasma creatinine, plasma cystatin C decreased during bedrest (1.07±0.09 mg/L at day0, 1.00±0.07 mg/L at day7 and 0.93±0.10 mg/L at day14, P<0.05 vs day0) indicating an up-regulation of glomerular filtration rate.

#### Correlation of changes in urea and creatinine with changes in anthropometrical indices

**Figure 3:**
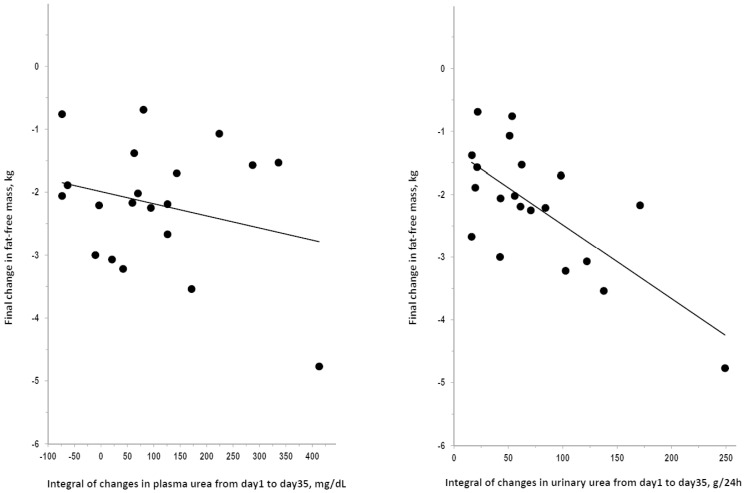
Correlation of changes in plasma and urinary levels of urea with changes in fat-free mass after bedrest. Plot of the after-bedrest final change in fat-free mass over the integral of changes from day1 to day35 of bedrest in plasma urea (R = 0.259, P = 0.270) and in urinary urea (R = 0.706, P<0.001). The correlation coefficient for the integral of changes in urinary urea was significant also after exclusion of the case with fat-free mass change below −4 kg (R = 0.494, P = 0.032) and with control for baseline values of the variables (partial R = 0.687, P = 0.002).

**Figure 4:**
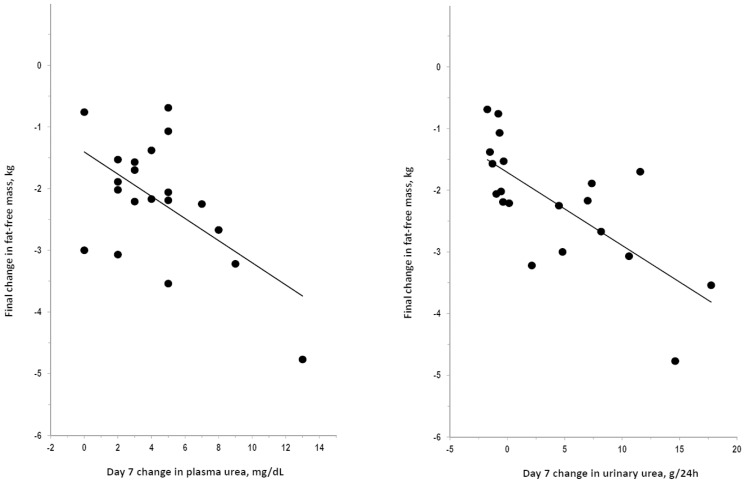
Correlation of day7 changes in plasma and urinary levels of urea with changes in fat-free mass after bedrest. Plot of the after-bedrest final change in fat-free mass over the day7 change in plasma urea (R = 0.566, P = 0.009) and in urinary urea (R = 0.715, P<0.001). The correlation coefficient for day7 change in plasma and urinary urea were significant also after exclusion of the case with fat-free mass change <4 kg (R = 0.554 and 0.640, P<0.01) and with control for baseline values of the variables (partial R = 0.608 and 0.684, P<0.01).

The after-bedrest final change in weight did not correlate with the integral of changes from day1 to day35 in plasma urea (R = 0.308, P = 0.186) or in urinary urea (R = 0.084, P = 0.726). The after-bedrest final change in fat-free mass did not correlate with the integral of changes from day1 to day35 in plasma urea but significantly correlated with the integral of changes from day1 to day35 in urinary urea (Figure 3). Findings were similar for fat-free mass and muscle mass because the after-bedrest final change in muscle mass did not correlate with the integral of changes from day1 to day35 in plasma urea (R = 0.339, P = 0.144) but significantly correlated with the integral of changes from day1 to day35 in urinary urea (R = 0.661, P = 0.002). The after-bedrest final changes in fat-free mass were significantly predicted by the day7 change in plasma urea or urinary urea (Figure 4). The day7 change in plasma urea or urinary urea significantly predicted also the after-bedrest final change in muscle mass (R = 0.451 and 0.513, respectively; P<0.05). Findings were never statistically significant in any analysis on the correlation of the after-bedrest final changes in weight, fat-free mass, or muscle mass with changes in plasma or urinary creatinine (R≤0.37, P>0.10, not shown). Findings were similar in correlation analyses focusing on data of specific days or of specific intervals of bedrest (data not shown).

## Discussion

This study analyzed in 20 healthy young men the bedrest-associated changes in indices of urea and creatinine metabolism. In addition to the expected decreases in plasma volume, weight, fat-free mass, and muscle mass, bedrest associated with transient increases in plasma and urinary urea, with decreased plasma creatinine, and with unchanged urinary creatinine. Correlations with changes in fat-free mass and muscle mass were significant for changes in plasma and urinary urea but not for changes in plasma or urinary creatinine. Moreover, changes in plasma and urinary urea in the first week of bedrest were significant predictors of final changes in fat-free mass and muscle mass after 35-day bedrest.

Urea is the catabolite of dietary protein or endogenous protein and is rapidly excreted into urine after generation [6]. Disorders associated with dehydration/hypovolemia or with hyper-catabolism increase plasma urea [24], [25]. Thus, the bedrest-induced reduction in plasma volume likely contributed to increase plasma urea. This contributory role diminished after day7 when hypovolemia was less noticeable but plasma urea was still high. The parallel time-courses of the increases in 24 h urinary urea and in morning plasma urea supported the interpretation that bedrest associated with an increase in urea generation because high urinary urea excretion without high urea generation would have decreased rather than increased plasma urea. The high urea generation during bedrest could reflect only an accelerated catabolism of endogenous protein because overall calorie intake and protein intake were reduced during bedrest as per study protocol. Urinary urea re-trended toward baseline after day28 suggesting that the catabolic phase progressively had slowed down at that time as indicated also by the finding of lower rate of mass reduction after day21. A reasonable explanation for the earlier decline in plasma urea could be that plasma urea was lowered by an accelerated urea excretion through the kidney because the stable decreases in plasma creatinine and plasma cystatin C pointed to a stable up-regulation of glomerular filtration rate after day7 of bedrest. In accordance with these interpretations, the observation of unchanged urinary creatinine in the presence of a stable reduction in plasma creatinine after day7 indicated that bedrest accelerated the renal excretion of creatinine but had negligible effects on creatinine generation. In turn, the lack of sizeable changes in urinary creatinine indicated that the mass changes associated with 35-day bedrest did not affect the muscular energetic metabolism.

Bedrest was followed by a weight loss almost exclusively due to a loss in fat-free mass which, in turn, was partly accounted for by a loss in muscle mass. Study results prove that, under the same experimental conditions, there was a large inter-individual variability in the bedrest-associated changes in body mass because the losses of fat-free mass and muscle mass ranged from zero to several kilograms. The correlation of the integral of the overall changes in urinary urea during bedrest with the losses in muscle mass and fat-free mass suggested that the sites of increased protein catabolism were skeletal muscle and other lean body compartments. Finally, the lack of correlations for the integral of the changes in urinary or plasma creatinine was in accordance with the conclusion that bedrest did not affected the muscular energetic metabolism which is the main determinant of creatinine generation [7].

Study results are in accordance with the repeated observations of bedrest effects on plasma volume, weight, fat-free mass, and muscle mass [1]–[5] as well as with the limited information about changes in urea or creatinine metabolism during bedrest [8]–[13]. The study does not support the findings of Millet et al. who concluded that bedrest associated with increased urinary urea excretion in women but not in men [11]. To the best of the authors' knowledge, this is the first report of a correlation between changes in urea metabolism and changes in body mass during bedrest.

The main limitations of the study were the lack of assessments for different body compartments more accurate than bioimpedance, the lack of data about total nitrogen, fecal nitrogen, and recovery after bedrest completion. The possibility of a bias in analyses on muscle mass changes cannot be excluded due to the lack of assessments by dual-energy X-ray absorptiometry or computed tomography [26]. This limitation should be absent or less important in analyses on fat-free mass given that bioimpedentiometric methods are validated for fat mass and fat-free mass [27]. The lack of data about non-urea nitrogen should have played only a minor confounding, if any, given that urinary urea accounts for the large majority of urinary nitrogen and is a reliable index of urinary total nitrogen [28]. Finally, the lack of fecal data was an unlikely cause of major bias because the intestine accounts for a minority of urea excretion [6] and for a negligible fraction of creatinine excretion [7].

The practical implications of study results are about the use of plasma or urinary urea as markers of protein catabolism and body mass losses in the presence of hypokinetic conditions as bedrest. These implications appear theoretically valid in the field of general medicine for patients undergoing prolonged hospitalizations as well as in the field of aerospace medical research. Actually, study results support also the use of plasma or urinary urea as predictors of body mass losses during bedrest because changes in plasma urinary or urinary urea after 7 days of bedrest significantly predicted the final changes in body mass after the completion of the 35-day bedrest.

In summary, in 20 healthy young men, a 35-day bedrest induced a transient increase in urinary and plasma levels of urea in addition to the expected reductions in some anthropometrical indices. The increases in plasma and urinary levels of urea appeared as the result of an increased urea generation secondary to the catabolism of endogenous protein of skeletal muscle and fat-free tissues. The increase in urea generation correlated with and predicted the magnitude of the reduction in anthropometrical indices supporting the use of urea as a marker of hypokinesia-induced catabolic conditions.
